# The geography of pre-criminal space: epidemiological imaginations of radicalisation risk in the UK Prevent Strategy, 2007–2017

**DOI:** 10.1080/17539153.2017.1327141

**Published:** 2017-06-01

**Authors:** Charlotte Heath-Kelly

**Affiliations:** ^a^ PAIS, University of Warwick, Coventry, UK

**Keywords:** Prevent strategy, radicalisation, pre-crime, pre-criminal space, counterterrorism, risk, pre-emption

## Abstract

This article explores geographical and epistemological shifts in the deployment of the UK Prevent strategy, 2007–2017. Counter-radicalisation policies of the Labour governments (2006–2010) focused heavily upon resilience-building activities in residential communities. They borrowed from historical models of crime prevention and public health to imagine radicalisation risk as an epidemiological concern in areas showing a 2% or higher demography of Muslims. However, this racialised and localised imagination of pre-criminal space was replaced after the election of the Conservative-Liberal Democrat coalition in 2010. Residential communities were then de-emphasised as sites of risk, transmission and pre-criminal intervention. The Prevent Duty now deploys counter-radicalisation through national networks of education and health-care provision. Localised models of crime prevention (and their statistical, crime prevention epistemologies) have been de-emphasised in favour of big data inflected epistemologies of inductive, population-wide “safeguarding”. Through the biopolitical discourse of “safeguarding vulnerable adults”, the Prevent Duty has radically reconstituted the epidemiological imagination of pre-criminal space, imagining that all bodies are potentially vulnerable to infection by radicalisers and thus warrant surveillance.

## Introduction

In studying the counter-radicalisation practices which constitute the UK’s Prevent strategy, academics have explored the deployment of pre-crime interventions, targeted disruption, rehabilitation, and risk assessment upon individuals and groups (Elshimi 2015; De Goede and Simon 2012; Gutowski 2011; Heath-Kelly 2013; Kundnani 2009; Lindekilde 2012). The Prevent strategy is, as the name suggests, a series of preventative measures used against persons thought to be at higher risk of becoming terrorists, or showing signs of “extremism” (understood by the Home Office as the ideological and behavioural precursor to political violence). This article traces the imagination of radicalisation risk in UK government policy since the rushed emergence of Prevent after the 2005 London bombings. It argues that shifts in the Prevent strategy reveal the reconceptualisation of pre-crime in British counterterrorism.

Initially, the Prevent pathfinder programme of 2007–2008 performed pre-criminal intervention in the form of community engagement activities and community policing, undertaken by local authorities in seventy areas considered high-risk by the Office for Security and Counterterrorism (OSCT) (Local Government Association 2008, 2; Thomas 2012). But this localised geography of pre-criminal intervention was de-emphasised (not abolished) in 2010, once the Conservative and Liberal Democrat coalition government took power and commissioned the Prevent Review. In the context of their economic austerity agenda, the implementation of Prevent through the Department for Communities and Local Government ended. Prevent delivery was centralised in the OSCT, ostensibly to simplify its delivery and reduce the allocation of funding for community engagement activities. Labour’s localised imaginations of extremism risk in residential communities were then side-lined by the government’s new “Prevent duty”. This new policy imagines extremism risk *nationally*, inserting “radicalisation awareness” into already existing national structures for safeguarding vulnerable adults^1^ in health care and schools.

In both eras of counter-radicalisation, the prevention of terrorism was understood to operate in an explicitly “pre-criminal space” – a phrase that occurs four times in NHS England’s *Prevent Training and Competencies Framework* (Goldberg, Jadhav, and Younis 2016; NHS England 2015). This term is left largely undefined, standing as an empty, but central, signifier in the governmental discourse of preventative (yet banal) intervention upon lives rendered simultaneously risky and vulnerable-to-becoming-terrorist (Heath-Kelly 2013a). But how is pre-criminal space constituted in the imagination of policy-makers, and how have crime prevention programmes been adapted to deliver this form of counterterrorism?

Pre-criminal measures trace their ancestry to crime prevention measures of the mid-twentieth century which used data on prior criminal conduct, school drop-outs and economic deprivation to model the probability of future offending (McCulloch and Wilson 2016, 9). An area’s potential to experience crime became predictable and calculable through statistical analysis. As I will show in the next section, crime prevention was imagined through local geographies, statistical data and the calculative rationality of risk. In the 1990s, criminal justice embraced the predictive turn, shifting further away from traditional models of retrospective intervention by bringing forward the threshold of criminal responsibility. Acts undertaken *in preparation for* criminal offending, or anti-social conduct imagined as prelude to future offending, became punishable in-and-of-themselves (ibid., 9–25).

The practice of pre-criminal justice as pre-emption develops from these earlier statistically oriented models. In the context of the securitisation of crime, and the War on Terror, pre-crime has since introduced a specifically anticipatory form of policing. In the early 2000s, US and UK police were awarded new powers to pre-emptively detain suspects in counterterrorism investigations for significant periods of time. As McCulloch and Wilson point out, pre-crimes are crimes which have not happened and are not imminent (ibid., 25). With no crime scene evidence or materials demonstrating preparation for a criminal act, the evidence underwriting arrest is replaced by the role of suspicion. If police intelligence places a suspect on a nascent trajectory towards terrorist offending, or if one’s travel data or financial transactions trigger the digital systems which monitor for flagged behaviours or deviations from standard patterns (Amoore and Marieke 2005; De Goede, Marieke 2012), then pre-emptive intervention can occur.

The politics of knowledge which construct trajectories of extremism and radicalisation are vastly important to the performance of pre-criminal intervention under the Prevent Strategy. This article explores how imaginative geographies of extremism risk have changed throughout 10 years of the Prevent Strategy, outlining the shift from Prevent’s localised pre-criminal intervention in residential neighbourhoods to the national roll-out of the Prevent Duty. The 2015 Prevent Duty invoked a nationalised imagination of pre-criminal space. It appropriates national structures of education and healthcare to apply preventative surveillance to all citizens, for their own protection (Home Office 2015b). It calls this “safeguarding vulnerable adults” against terrorism. The Prevent Duty thus securitises *all* bodies as potentially vulnerable to contamination by extremism (even if Muslim and brown bodies still make up the majority of referrals made to the police).

The nationalised imaginary of extremism risk in the Prevent Duty deviates from traditional surveillance and profiling – which begins its work by imagining a defined suspect group. Even if the Prevent Duty ends up targeting brown bodies, its calculative rationality does not begin by imagining them as the location of radicalisation risk. Instead, the nationalised Prevent Duty replicates a big data logic found in the digital arena – it prioritises the scale of a vast sample size, rather than beginning from the suspect community imaginary we have come to expect from counterterrorism programmes (Hillyard 1993; Pantazis and Pemberton 2009; Ragazzi 2016). But, because schoolteachers and health-care professionals are not immune to Islamaphobic media discourses, they apply their duties of suspicion unequally and replicate the stigmatisation of brown bodies found in Labour’s Prevent Strategy.

In this way, the performance of pre-crime in Prevent has moved towards algorithmic logics of detection and inductive profiling (Heath-Kelly 2016). A calculative shift has occurred which imagines pre-criminal space nationally, and Prevent’s deployment has been reorganised and extended to fit this landscape.

## British governments and their approaches to counter-radicalisation

The Prevent agenda was first invented by Tony Blair’s government in 2006. It was the first example of a counter-radicalisation strategy in Europe or America. It was pre-existed by the de-radicalisation interventions of the Saudi Arabian regime which has, since 2004, attempted to un-teach “deviant” interpretations of Islam through religious re-education in prisons (Boucek 2008). Yet, Saudi Arabia’s rehabilitation of prison populations is an example of criminal prevention rather than pre-crime intervention; it relies on statistical data about offending rates to predict future crime risk (McCulloch and Wilson 2016). It utilises the *statistical calculative rationality* of crime prevention. This makes the UK’s counter-radicalisation strategy the first to take on an explicitly *pre-crime* formulation of prevention (as opposed to statistically targeted rehabilitation measures). Without the use of statistical data on offending rates, Blair’s Prevent strategy imagined a pre-criminal space within British Asian residential areas where extremism risk necessitated intervention.

The originality of the Prevent Strategy must be understood within the context of the London bombings of 2005 and the failed tube bombing which immediately followed. The government was thrown into a panicked search for methods to prevent suicide bombers who, unlike previous generations of militants, possessed few links to established organisations. Traditional surveillance, suppression and infiltration techniques from the campaign against the Provisional Irish Republican Army (IRA) could not be replicated. In this knowledge and policy vacuum, the discourse of radicalisation emerged to explain the seemingly individualised and disconnected pathways of citizens into armed militancy (Heath-Kelly 2013a; Kirby 2007; Sageman 2004; Sedgwick 2010). In response to this new discourse of decentralised threat, Blair’s government introduced the Prevent strategy as an anticipatory programme of counter-radicalisation, deployed by local authorities to identify and counter extremist influences.

Early Prevent documentation explicitly framed counter-radicalisation as deploying community cohesion and moderate Islam against extremist influences, to bring about preventative effects through community resilience:

It is not for government to intervene in theological debates, but there is a role for government in […] providing effective campaigns to confront extremist ideologies; promoting local role models able to counter negative imagery and comment; promoting understanding of the benefits that Muslims have brought to local areas; promoting understanding and acceptance of key shared values, and promoting dialogue and engagement between communities in support of those values. (Department of Communities and Local Government 2007, 5)

The Department for Communities and Local Government oversaw the initial Prevent “pathfinder” pilot of 2007/8 which targeted intelligence gathering and community engagement activities at 70 local communities mapped as high-risk for producing extremists (Department of Communities and Local Government 2007; see the Appendix for the 70 priority areas defined by central government as high risk in 2007). The demographic make-up of areas funded by the Prevent Pathfinder programme in 2008–2009 suggests that their “extremism risk” and level of funding was directly constituted in relation to their numbers of Muslim residents (Kundnani 2009, 13–4).

As I will show in following sections, early Prevent operations under Labour governments redeployed the rationales of public health and criminal prevention models from the nineteenth and twentieth centuries. Here, communities were profiled as sites for health and policing interventions according to their statistically generated risk score for vulnerability to diseases or crime. The public health and pre-crime typologies of primary, secondary and tertiary preventative interventions were directly carried over into Labour’s Prevent strategy, constituting a heat-map of vulnerability to extremism based upon community demographics.

However, this did not last. Under the Coalition and subsequent Conservative governments, the apparatus and operations of counter-radicalisation have shifted. While capacity building within communities and community policing still occurs, Prevent planning and implementation has been considerably centralised in the Home Office since 2010. Local authorities have been cut out, now existing only as bidders for Home Office created Prevent activities and funds for their implementation. Furthermore, the new Prevent safeguarding duty^2^ is not applied exclusively to/through British Asian residential communities, but through the national systems of education and health care.

Of particular interest here is the 2011 Prevent Review undertaken by the Coalition government. The Coalition took office in 2010, after 13 years of Labour Party governments. Labour had previously implemented the Prevent Strategy through the Department for Communities and Local Government (DCLG), relying upon methods and assemblages previously used to deploy “community cohesion” (interventions designed to effect cross-community reduction of tensions) (Thomas 2012; Thomas 2014). The administrative geography of Prevent under Labour was heavily associated with local authority delivery of workshops and events that promoted “moderate Islam” in residential communities; Prevent governed through community, if you will.

The Coalition introduced a raft of austerity measures to reduce public spending, including the severing of Prevent’s delivery through the DCLG – removing the link to integration work and ownership of Prevent work by local authorities. The Prevent review centralised control of Prevent delivery within the Office of Security and Counter-Terrorism (OSCT) (Thomas 2014). Indeed, it de-emphasised “community” as the landscape for counterterrorism and moved Prevent towards administration through *whole-of-population* institutions (schools, universities and health-care premises). While “community” has not totally disappeared as the mechanism through which Prevent is delivered and articulated, and local variations exist in Prevent-funded community development (Therese et al. 2016), residential community has been deemphasised in favour of nationalised imaginations of terrorism risk and pre-criminal intervention.

As I will show, this is unusual. Prevent now embeds the reporting of deviance in national organisations because they have a high level of public contact. It defends this massively increased surveillance of the population by arguing that larger sample size is beneficial to counterterrorism. This logic is alien to crime prevention models which used statistical data to allocate risk scores to discrete areas. As such, post-2011 Prevent reflects an epistemology more common to big data and algorithmic tools.

Digital methods and epistemologies prioritise the collection of huge datasets and utilise computerised techniques to partition and reassemble the data scraps. Rather than reducing sample size and narrowing down onto suspect groups, the epistemology behind complexity science invokes the potential *within* huge data sets. The human eye cannot detect patterns at this scale, but complexity epistemology advocates that machines can identify correlations which have previously remained hidden (Amoore and Piotukh 2016). While the Prevent strategy does not utilise algorithms as such, the reworking of its administration and deployment suggests an influence of big data epistemological discourse on planners and policymakers (Heath-Kelly 2016) – one which has played a role in Prevent’s shifting geography of pre-crime.

While the epistemological and geographical imagination of extremism risk within Prevent is shifting towards nationalisation, I do not want to suggest that all ethnicities are equally made suspect by Prevent as a result. This article does not claim that white British and British Asians find themselves equally exposed to suspicion or intervention. Rather, it models the shift towards whole-of-population subjection to, and mass responsibilisation for, the Prevent Strategy. Prevent no longer begins from the assumption that pre-criminal interventions should target particular residential areas, even if its nationalisation still disproportionately stigmatises and affects British Muslims. An epistemological and geographical shift has occurred in the imagination and pre-emptive mapping of extremism risk.

## An epidemiology of radicalisation under labour

Pre-crime interventions have a long history. They did not begin as counterterrorism tactics, but have historically taken the form of crime reduction initiatives deployed upon “high-risk” areas and cases. And these crime reduction initiatives themselves rely upon prior discourses and methods of public health and preventative medicine. Antecedent public health strategies of the nineteenth and twentieth centuries produced geographical epidemiologies of threats to population health such as cholera. Importantly, these public health geographies utilised a tripartite frame to categorise the population, one which was directly carried over into pre-crime interventions and counter-radicalisation (of the New Labour era, 1997–2010).

At the “primary” level, preventative public health measures target the health of the *general population* vis-à-vis a threat or contaminant (rather than the sick, or a group thought highly likely to become sick). The general conditions which enable disease are the object of intervention. For example, a “primary” measure includes the introduction of a sewer system to manage human waste, thereby acting at the level of the entire population to prevent illness. “Secondary” preventative measures are localised interventions performed upon individuals and groups *showing early symptoms of disease*, or *at high likelihood* of contracting illness. This is a more focused intervention aimed at prevention contagion within particular communities. Finally, “tertiary” prevention is directed towards those *already suffering from a disease* – to cure them, rehabilitate them and prevent reoccurrence (Brantingham and Faust 1976; Van Dijk and Jaap 1991).

This public health geography of prevention is thus constituted around notions of proximity and contagion. Those already sick are found at the centre, whereas secondary health interventions pre-emptively target those deemed to be in proximity to contagions, and primary interventions intervene upon general enabling conditions at the level of the population.

These public heath typologies were directly transplanted from epidemiology to crime prevention efforts of the mid-twentieth century. Crime became something statistically predictable, inherently geographic and preventable in policy terms. As in public health, the primary level of crime prevention identified the general conditions that enable crime to occur. Primary crime prevention measures were then applied to the general population – whether or not they were deemed likely to become criminals or victims of crime – introducing neighbourhood watch policies and televised crime awareness campaigns (Van Dijk and Jaap 1991). The lacking awareness of crime in the broader population was here constituted as an enabling condition of crime.

The secondary level of crime prevention engaged in the early identification of potential criminals. It generated pre-crime demographics from statistical modelling of population data. Geographies of future offenders were produced from school drop-out figures, the locations of pockets of economically disadvantaged and untrained youths, and sometimes even the presence of citizens with physical and mental disabilities (Brantingham and Faust 1976). This particularly offensive symptomology is drawn from 1960s’ crime prevention initiatives in the United States, but all secondary crime prevention produces knowledge by analysing the statistical correlates of crime (school exclusion, economic disadvantage) and then reversing the analytic temporality – to assume that the correlative symptom *increases the probability of* crime. This generates a risk score.

In such correlative analysis, an epidemiological geography of crime was created – a map of supposed pre-crime areas and demographics. These spaces were then targeted with job training, education interventions and social activities which supposedly promoted resilience and civic responsibility.

Finally, tertiary prevention was transplanted from the already-diseased of public health to the convicted offenders of criminal justice. Even this tertiary level was considered preventative (rather than simply punitive), because the judicial response to crime aimed at the reduction of recidivism through the separation of offenders from the population (imprisonment), rehabilitation programmes and treatment programmes for addiction (Van Dijk and Jaap 1991).

The three levels constitute a geographical model of offending risk and criminal prevention through statistics. The calculative rationality underpinning public health interventions and “criminal prevention” is the statistical paradigm which emerged in the nineteenth century. This model replaced Enlightenment faith in human nature, natural law and determinative causality (Hacking, Ian 1990). In the statistical paradigm, *probabilities* are used to calculate the chance of future disorder or illness and then to direct appropriate political interventions. Probability science involves abstracting from the individual case to the level of all cases. Through the collection of crime figures, poverty statistics and school-leaving data, base rates of statistical probability (“regularities” within a large population) can be produced. This produces knowledge about likely futures. This knowledge production is simultaneously a mapping exercise – creating a geographical model of risk and pre-criminal intervention.

The same epidemiological typology of prevention can be directly mapped onto the early years (2007–2011) of the UK Prevent Strategy. Under the Labour governments of the early twenty-first century, counterterrorism prevention was (hurriedly) deployed through the geography of primary, secondary and tertiary interventions, constituting an imagined, epidemiological geography of extremism risk.

One of the first official steps in terrorism prevention involved the increased responsibilisation of the general public for preventing terrorism. In the style of *primary* pre-crime interventions upon the general “enabling conditions” of crime, the public were tasked with reporting suspect packages and behaviour to the police through public awareness campaigns. Posters and tannoy announcements adorned the walls and filled the concourses of public transport networks, instructing citizens to report any suspicious activity or packages to the British Transport Police. Coaffee and Rogers (2007) have described this as the post-September 11 intensification of previous emergency planning doctrines, where individual citizens are now called upon to play a role in urban risk management. Coaffee’s work also points to governmental perception that openness, as a condition of unfettered movement within cities, enables terrorism to occur. In this geographical reading of threat mobility, governments turn to material objects like “rings of steel”, bollards and cordons to protect financial districts and parliaments (Coaffee 2004).

In this conceptualisation of primary-level terrorism prevention, the general conditions which enable crime to occur are the non-suspicious attitude of the population and the relative openness of the urban environment. To combat the threat of terrorism, both population and urban infrastructure are adapted to disrupt the malicious intents of deviants. These primary-level preventative measures were also used by the British Government with regard to the IRA campaign.

After these primary-level interventions in the domestic arena failed to prevent the London bombings of 2005, the British government adopted the secondary and tertiary levels of pre-crime intervention – making their innovative leap into pre-crime. Policy-makers and media constituted the “radicalisation” discourse to explain how, and why, British nationals would bomb London. The discourse relies upon the imagination of a (disruptable) socialisation process of peer-pressure and ideological change which leads to terrorism – a model very different to previous research in Terrorism Studies on protest movements and root causes.^3^ The individualised discourse of radicalisation emerged immediately after the London bombings (Kundnani 2015; Sedgwick 2010), and imagined the process by which British citizens could turn against their own government, under the influence of “extremist ideology” and disenfranchisement.

Counter-radicalisation interventions were aligned with the secondary level of prevention under the Labour governments: they mapped those considered *likely to develop* symptoms of terrorism. But what statistical data could be used to model the chance of terrorists emerging in a given area? Unlike public health interventions, imaginative geographies of radicalisation risk struggle to generate predictive statistical models. As Mark Sageman (2016) has convincingly argued, cases of terrorism are too few and far between to construct the base rate upon which a statistical model relies. Unlike the predictive modelling of burglary and street crime, there are not enough terrorists to produce statistical models of terrorism and allocate risk scores to areas. In this vacuum, residential demographics substituted for the statistical base rate of previous crime prevention models.

In 2007, Prevent funding was provided to seventy local authorities in relation to the numbers of Muslims in their area. If an area had been 5% comprised of Muslims in the 2001 census data, or more, then it qualified for the new Prevent funds (Thomas 2014). This threshold was later reduced to 2%, according to Paul Thomas (ibid.). This funding-according-to-demographic clearly demonstrates the reductive and offensive association of Muslims and British Asian communities with extremism potential by central government. The number of Muslims was understood as a precursor to terrorism by the government, given the stated faith of the 9/11 hijackers and the 7/7 London bombers. The demographic of residential communities became constituted as the secondary level of intervention – the “at-risk” population requiring preventative intervention.

Prevent funding from central government was used to fund a wide variety of social and training activities for British Asian residential communities, including language courses, training in accessing local services, sports, and cross-community work – operating in parallel with assemblages of “community cohesion” (Kundnani 2009; Thomas 2010). Policies of “community cohesion” had been introduced after riots in British Asian areas of Northern cities in the summer of 2001. Despite these riots being provoked by white nationalist agitators, the Cantle report on the disturbances advocated preventative measures which would (among other things) reduce community self-segregation. To build cross-community engagement, community cohesion policies funded local “shared events”, as well as “arrival packs” for new residents (Kundnani 2009; Worley 2005). The distribution of Prevent funding to local authorities according to population demographics, to projects mirroring community cohesion assemblages, saw the creation of a Prevent geography where race (even if not mentioned by name) constituted the key condition for potential contagion. Prevent mapped British Asian communities as being “at risk of becoming risky” (Heath-Kelly 2013) in the style of public health interventions on communities thought likely to become sick. Prevent then deployed local organisations to build resilience to extremism in these areas and trained favoured “moderate” figures to this end.

The original “Preventing Violent Extremism” documentation was reasonably open about the government’s perception of British Asian communities as spaces of ideological conflict and vulnerability, advocating the promotion of moderate voices against extremist narratives (Department of Communities and Local Government 2007). The funding of educational and social activities through a counterterrorism remit was intended to build the “resilience” of these communities to violent extremism – increasing their resistance to problematic ideologies and enabling them to challenge extremist viewpoints (Home Office 2009).

The language of resilience as resistance to extremist ideologies directly parallels the language of contagions and the fostering of immunity. Unlike community cohesion work, however, voluntary organisations and local authority staff working with Prevent money soon became uncomfortable with the expectation to pass information about communities and individuals to the police (as well as the embedding of counterterrorism officers in service delivery). The national government’s initial attempt to fund community cohesion work and Prevent work in equal measure steadily gave way to the dominance of securitising actors and perspectives at both local and national levels (Thomas 2014). In Birmingham, for example, the introduction of Prevent saw a leading Counterterrorism Unit officer transplanted into the Council Equalities Division to manage the project funding – causing great alarm about the implicit surveillance of communities being undertaken (Therese et al. 2016). In the interaction between national and local governments, the Home Office applied pressure upon local authorities to adopt Prevent reporting measures (national indicator 35) and tip their practice towards monitoring British Asian communities rather than community cohesion activities (Thomas 2010).

The congruence of surveillance with the funding of community events should not surprise us, once we note the translation of the tripartite model of criminal and epidemiological prevention. Once communities are mapped as locations of secondary contagion likely to develop symptoms, even well-intentioned activities dedicated to capacity building and resilience are situated within the securitised prevention of deviance. Underneath the ambiguous language of “community” and the fluffy funding of sports lurked a very real governance agenda of secondary crime prevention designed to intervene upon those considered likely to develop symptoms of radicalisation. Surveillance is the obvious counterpart to community integration in this model.

In the Labour era, one might describe Prevent’s “secondary-level interventions” as the meeting point between “community cohesion” (which promoted integration and cross-community relations through voluntary organisations) and “community policing”. Both operate at the secondary level of crime prevention, targeting those groups who have been designated as vulnerable to future disorder through statistical modelling exercises. Community policing is an approach to law enforcement which maximises collaboration with local residents, responding to neighbourhood grievances in exchange for the facilitation of flows of information about crime. It constitutes areas of pre-crime through statistical modelling and then embeds officers in the community to reduce the threat of potential deviance. This type of policing originally had roots in the prosecution of neighbourhood crimes but was adapted to counterterrorism in post-7/7 Britain (Klausen 2009). As Klausen argues, policy-makers favoured bolstering the relationship between British-Asian communities and police to both improve the potential flow of counterterrorist intelligence and also mitigate any damage resulting from operations and arrests.

Such national policy on counterterrorism had the effect of constituting Muslim neighbourhoods as both “stakeholders” in and suspects of the Prevent agenda. They were implicitly portrayed as both at-risk (and deserving of additional resourcing and support) and risky (to be surveilled as potential threats) (Heath-Kelly 2013). Furthermore, this command that communities engage with the state, via their local police, produced binary identities *within* communities on the basis of co-operation. As Basia Spalek and Alia Imtoual argue, community engagement practices produced binaries of legitimate/illegitimate, moderate/radical Muslims (Spalek and Imtoual 2007). The early geography of Prevent thus constituted British Asian residential neighbourhoods as locations of secondary intervention on its heat map of pre-crime (see Kundnani 2009 and the Appendix), before allocating privileged status to certain members of those neighbourhoods though their collaboration with prevention endeavours.

These dynamics speak to the second meaning of “community policing”, alluded to in recent work by Francesco Ragazzi as “policed multiculturalism”, whereby the policy performatively enacts and constitutes the “community” it names (Ragazzi 2015). Here, counterterrorism officers police the boundaries of the community through designations of who, and who does not, count as a relevant stakeholder. Simultaneously, however, community engagement is also a productive deployment of power; members of “engaged” communities self-nominate to take on roles in the adjudication and monitoring of conduct, as well as the promotion of a self-defined “moderate” Islam (Ragazzi 2016).

This perspective is an important corrective to the “suspect community” thesis which highlights the legal and cultural assemblages which constitute ethnic groups as potentially dangerous and subject to exceptional treatment (Breen-Smyth 2014; Hickman et al. 2011; Hillyard 1993). As Ragazzi, Spalek and Imtoual and other scholars show, the constitution of a community as suspicious is not simply a disciplinary action performed from outside; rather, collusory conduct is also induced from community members who are awarded privileged status in exchange for cooperation. But whatever the specificities of counterterrorism dynamics in communities rendered suspect, there can be little doubt that Prevent’s early geography conforms to a secondary-level pre-crime model. Communities identified as more vulnerable to becoming deviant were made subject to pre-crime interventions of capacity building, integration workshops and surveillance (Kundnani 2009). The epidemiological model fits.

Finally, the tertiary level of British counterterrorism under Labour acted upon those *already* made “radical”. To refer back to the original conceptualisation of tertiary crime prevention, it intervenes upon convicted offenders to reduce their reoffending risk through long-term incarceration and/or rehabilitation (Brantingham and Faust 1976). In a further study of the translation of public health models to crime prevention, van Dijk and Jaap confirmed that tertiary crime prevention acts upon offenders and ex-offenders through rehabilitation and treatment programmes (1991). This tertiary level of crime prevention also maps onto the Saudi deployment of theological corrections upon prisoners, to prevent future offending.

In Brantingham and Faust's (1976) terms, the tertiary dimensions of British counterterrorism under the Labour governments were demonstrated in the open-ended detention of terrorism suspects under control orders and TPIMs.^4^ These house-arrest programmes were efforts to prevent potential deviance through immobility. Their geography is largely a secret one. The artist Edmund Clark recently exhibited his work “Control Order House” at London’s Imperial War Museum, speaking directly to the proliferation of secretive residential detention (2013). Clark’s photographic exhibition explores the mundane, stripped bare existence of the residence under indefinite legal curfew. It conveys the silent worlds of such detention which exist secretly alongside us, in every town and city, mapped by the Home Office and Police but unknown to the population.

Other tertiary measures also existed and continue to exist. The Channel programme of multi-agency rehabilitation interventions for those showing signs of extremism has a similar tertiary geography of secrecy; like control order houses, its presence is ever-present yet unseen by the public. The Association of Chief Police Officers maintains organisational control over Channel – which is described as a multiagency collaboration between local authorities, educational partners, health services partners, social services, children’s services, police and offender management partners to “divert people away from the risk they face before illegality occurs” (Home Office 2012, 4). It is a de-radicalisation programme present in every local authority, focusing on the rehabilitation of those thought to be on the path towards terrorism through mentoring and counselling by an approved community peer.

But, unlike normal criminal rehabilitation of offenders, counterterrorism has *brought tertiary prevention forward in time*: into the pre-terrorist stage. Convicted terrorists are not made subject to Channel (they are imprisoned); rather, it is those categorised *as moving towards violent extremism* who are subjected to Channel interventions. This premature deployment of rehabilitation upon the potential offender speaks to the underlying temporal confusion of the radicalisation discourse: knowledge cannot identify the tipping point when illiberal thought and behaviour produces terrorism. In this grey-zone, Prevent performatively constitutes a new category of offender (Heath-Kelly 2013; Sageman 2016) – the pre-criminal “terrorist” requiring rehabilitation before they commit a crime. The epidemiology of radicalisation risk introduces the treatment stage before crime, or even criminal preparation, has occurred. The imagination of pre-criminal states brings forward the threshold of deviancy and intervention, enabling the “rehabilitation” of persons reported to police by community leaders for holding political or religious views deemed suspicious.

To conclude, under post-9/11 Labour governments, the deployment of pre-criminal interventions largely followed the tripartite structure of previous criminal and public health models of prevention – albeit, with racialisation replacing the statistical knowledge which underwrote previous eras of criminal prevention. Separate measures were deployed at each “level” of intervention: public information campaigns at the primary level of the general populous; community workshops and community policing at the secondary level of residential communities deemed vulnerable; and preventative-rehabilitation deployed upon those considered already affected by radical contagion at the tertiary level. The Labour governments translated criminal prevention and community cohesion models into counterterrorism, but reworked them around a different calculative rationality: statistical prediction was replaced by the imagination of pre-criminal space and epidemiological vulnerability attached to race. This endorsement of pre-emption and pre-crime was to become even more pronounced in the subsequent Coalition and Conservative eras.

## Nationalising pre-criminal space: prevent under the conservatives

Since the election of the Coalition government in 2010, the geographical application and the epistemology of the Prevent strategy has changed. Upon election in May 2010, all secondary-level Prevent work was immediately suspended pending the Prevent Review of 2011.^5^ This official review explored Labour’s counter-radicalisation structures, then permanently removed responsibilities for the Prevent Strategy from the Department of Communities and Local Government (DCLG) and instead passed them to the Home Office under the OSCT. The Coalition Government decided that previous Labour administrations had muddled the delivery of two separate areas of policy: community integration and counterterrorism (Home Office 2011, 1). This can be interpreted as a direct criticism of Prevent’s secondary level of crime prevention. The Coalition and Conservative governments’ geography of counterterrorism has largely de-operationalised residential communities in the delivery of Prevent, removing the DCLG from operations and reducing the number of local authorities funded to deliver Prevent activities from 70 to 28.

Alongside the continuation of limited community engagement activities, Prevent is now deployed through Research Information and Communications Unit (RICU) messaging from the OSCT and through whole-of-population institutions of health care and education. The Prevent Duty (the legal duty to report suspicions of radicalisation) has operated in all schools, health-care premises and universities since at least 2015, but in priority areas since 2011.

This has split the administration of the Prevent Strategy between localised community focused engagement (which works from an assumption about suspicious locales and identities) and the national level (which acts upon the whole of population). To some degree, particularly ethnicities are still imagined as pre-criminal and epidemiologically vulnerable, despite the wider roll-out of Prevent surveillance to all citizens. For example, RICU messaging has been deliberately targeted at particular ethnicities and age ranges thought to be higher risk for extremism. The RICU of the Home Office, founded in 2007 and led by Commander Steve Tatham, was previously responsible for psychological operations at the Ministry of Defence (Sabir 2017). After undertaking research projects on British Muslim communities and online habits, Sabir describes how the unit began deploying covert anti-radicalisation messaging towards young males of Pakistani, Bengali and Somali ethnicity. This messaging was delivered covertly by employing several PR firms (Breakthrough Media Network; Bell Pottinger) to disseminate the unit’s propaganda – obscuring the source.

Given the ethnic profiling undertaken by RICU, we can clearly see that the secondary level of pre-crime intervention has not been discarded altogether. Racial profiling still informs the delivery of preventative interventions and the supposed building of “resilience” to extremism. Furthermore, a reduced number of local authorities (70 reduced to 28) still receive some central government funding to deliver Prevent interventions and capacity building upon their populations (Thomas 2014). The government advises that intelligence agencies and police have identified these 28 areas as “hot spots” of the greatest vulnerability, but publicly available maps of their location are unavailable. We can, however, assume that the 28 hot spots are drawn from the 70 areas listed as high risk for extremism in 2007 though (Appendix).

Both the materials and funding for Prevent work are now drawn from central government sources, with local authorities effectively being paid to deliver Home Office content with little agency. A recent leak of Home Office documents regarding Prevent activities conducted in 2015 confirms this reading (Home Office 2015a). It shows that the central-government-funded local authorities and outreach organisations to deliver centrally produced content, including school plays about a Muslim Imam deployed with the British Army, the training of Muslim women in countering extremist rhetoric, and videos shown in schools and youth centres about boys who make bad choices and join jihadist or far right organisations. The 28 local authorities were used like local franchises for the delivery of the Home Office’s message.

The delivery of government workshops by youth workers and civil servants, within areas designated high-risk areas requiring secondary-level intervention, speaks to Francesco Ragazzi’s research on the securitisation of social policy in Western Europe during the War on Terror (Ragazzi 2017). In the delivery of these secondary interventions, and the tertiary performance of “rehabilitation” upon the radicalised by multi-agency panels (the Channel programme), we can recognise Ragazzi’s argument that social policy has been securitised. The securitising move relies upon the historical trust placed in social care professionals by populations, which is then used to introduce counterterrorism policing and surveillance-masked-as-social-care into suspect residential communities.

However, the model of securitised social work captures the Labour government’s Prevent Strategy better than it does the Conservative. While the post-2011 Prevent arena still deploys some elements of secondary-level intervention (identifying 28 high priority local areas for Home Office produced content, targeting particular ethnicities with RICU messaging), the remainder of the policy operates according to a different epistemological logic. Through the Prevent Duty, the secondary level of epidemiology (vulnerability to infection) has become blurred with the primary (the generalised conditions which enable the spread of disease within human populations).

The major development in the Conservative government’s Prevent Strategy has been the 2015 Prevent Duty (Home Office 2015b). This Duty has legally enforced a requirement on all schools, nurseries, health-care premises and universities to have “due regard” for preventing terrorism. All schools must be aware of radicalisation risks, report signs of radicalisation to their police contact and teach “British values” to the children. Health-care premises are also required to roll out Prevent training to all staff with safeguarding duties and to report signs of radicalisation to their police contact. The Prevent Duty Guidance specifies that

‘Safeguarding’ is the process of protecting vulnerable people, whether from crime, other forms of abuse or (in the context of this document) from being drawn into terrorist related activity. (Home Office 2015b, 21)

In one discursive move, protecting vulnerable adults from abuse has been merged with nationwide counterterrorism monitoring. This biopolitical imagination of pre-criminal space constitutes vulnerable bodies as potential terrorists. We are all now “vulnerable bodies” though, because one’s status as vulnerable is defined circularly by one’s adoption of extreme views. One must *already have been vulnerable*, prior to being flagged as a potential radical, otherwise such pathological views could not have developed. As such, the deployment of counter-radicalisation through the Prevent Duty blurs the primary and secondary levels of criminal prevention/public health intervention. *Every* community is vulnerable to extremist infection and criminal proclivity; the general epidemiological enabling condition is reworked as human existence itself.

At the moment, universities have lighter responsibilities under the 2015 Prevent Duty (Home Office 2015b) and are not required to train academic staff in the detection and reporting of radicalisation (although some choose to roll out the Government’s WRAP training). In the view of my own institution, each university is required to perform a radicalisation risk assessment, put structures in place to deal with any reports of radicalisation, have an external speaker vetting process and to give government Prevent training only to “key” members of staff (usually interpreted as the student residences, counselling and security teams). The Prevent Duty thus lurks in the background of student–institutional interaction, unlike its overt deployment within schools and health-care premises.

By July 2016, the Home Office confirmed that 550,000 doctors, nurses and teachers had received Prevent Workshop to Raise Awareness of Prevent training (Jeory and Cockburn 2016). The number of social sector professionals given responsibility for counterterrorism continues to rise with the prolonged roll-out of Prevent training – and one could make the argument that social policy has indeed been further securitised. However, in making that argument, one also needs to account for the geographical shift in the implementation of the Prevent strategy. The vast network of doctors, nurses, lecturers and teachers now incorporated into counterterrorism reporting are not service providers only to the ethnicities and residential communities made suspect in Labour’s era. They aren’t only implementing their training upon designated high-risk groups; rather, the mechanics of the Prevent Duty are present in the social services provided to the whole-of-population.

Of course, Muslims are still constructed as a suspect community by Islamaphobic media reporting and government statements (such as David Cameron’s “muscular liberalism” address of 2011). However, the developments of Conservative Prevent policy appear to be de-emphasising residential communities as a starting point for pre-criminal intervention, in favour of national Prevent delivery through social service providers. Prevent’s geographical application has changed, merging the previously distinct categories of primary and secondary implementation.

The Prevent Duty also represents a dramatic epistemological shift away from statistical calculative methods. Statistical models of crime prevention could never support such a whole-of-population roll-out, because actuarial rationalities subtract from general population data to identify specific locations of probable threat. Instead of isolating particular areas of contagion and intervention, the Prevent duty now prioritises the responsibilities of national social care facilities for counterterrorism surveillance. This has had the effect of massively increasing the number of referrals made to Channel. Police and local authorities encounter a far larger amount of data about extremism as a result of this geographical expansion. For example, the number of referrals made to the Channel de-radicalisation programme has skyrocketed since the introduction of the Prevent Duty. For example, between 1 January and 31 December 2015, 3955 people were reported to Channel – a massive increase on previous years, up 209% from the referral of 1281 people in 2014. The 3955 referrals in 2015 is more even than the sum of 3943 referrals made *in the preceding eight years* (NPCC n.d.)!

These dramatic increases were the result, and apparently the intention, of the new Prevent geography. Indeed, the revised Prevent strategy describes schools and clinics as potential assets for counterterrorism because of their huge numbers of contacts with the population:

1.3 million NHS workers have contact with over 315,000 patients daily and some 700,000 workers in private and voluntary healthcare organisations see many thousands more […] *Given the very high numbers of people who come into contact with health professionals in this country, the sector is a critical partner in Prevent*. There are clearly many opportunities for doctors, nurses and other staff to help protect people from radicalisation. The key challenge is to ensure that healthcare workers can identify the signs that someone is vulnerable to radicalisation, interpret those signs correctly and access the relevant support. (Home Office 2011, 83–5; emphasis added)

This prioritisation of scale is indicative of a move from actuarial (statistical) modelling towards an algorithmic or big data rationality. As such, the geographical shift in Prevent is also an epistemological one. In the digital realm of inductive calculation, data is *not* used to calculate probability. Induction works according to different logics to produce imaginations of risk. Louise Amoore’s work on the UK e-borders programme has been pivotal in exploring algorithmic security calculation, showing how digital analytics combine discrete and unrelated pieces of data (like travel histories and methods of payment) to constitute possible futures of risk or normality (Amoore 2011). These data “scraps” generate patterns and correlations. The mode of inductive calculation associated with algorithmic prediction involves the ingestion, partitioning and machinic reassembly of vast amounts of data – transforming them into knowledge “spoken” into being by the algorithmic process (Louise and Piotukh 2015). Complexity becomes the modality of calculation, because it is presumed that such heterogeneity (on such a vast scale) has the potential to reveal new patterns and connections between previously disparate factors (Comfort et al. 2010; Heath-Kelly 2016).

Despite the usual restriction of algorithmic rationalities to the digital realm, there are reasons to believe that big data discourse has informed the geographical shift in Prevent strategy interventions, within the context of the Conservative austerity drive. First, the deployment of Prevent through the entire social services network renders the whole-of-population as an object of surveillance, despite no fears that the entire British population is about to rebel. Scale has been prioritised, and referral numbers have rocketed. Scale has become a modality of pre-crime intervention.

Second, the training given to social professionals does not train them to recognise a static risk profile of radicalisation symptoms. As I have shown elsewhere, the training is confused, vague and does not provide a profile of the radicalised subject (Heath-Kelly 2016). Indeed, NHS policy on Prevent emphasises that a static risk profile would not capture the nebulous and shifting character of radicalisation and thus staff must instead use their “professional judgement” to detect terrorists (NHS England 2015). This is interesting because it suggests the adoption of an inductive modality common to big data epistemologies. Here, prevent surveillance is understood to *generate* the terrorist profile rather than responding to it. Through prolific contacts with the public, social professionals are thought able to distinguish the radical from the normal. In this paradigm, their embeddedness in vast flows of contact (data) renders them as potential counterterrorism assets, capable of organically noticing the future radical (despite a constantly shifting profile) and alerting the police.

The geographical shift in Prevent is thus accompanied by altered epistemological commitments. The prioritisation of scale, and the refusal to limit the remit of Prevent to the reporting of defined symptoms, indicates a non-actuarial calculative rationality. Vast swathes of social workers are expected to intuit the presence of radical tendencies, rather than to report profiled behaviours.

Indeed, if Prevent were still embedded in a statistical calculative rationality, it would have been discarded as a failure. In 2016, 90% of NHS referrals to Channel were assessed by the Police as not related to terrorism or extremism. The referrals were instead reclassified as requiring other types of safeguarding intervention (housing, drug and alcohol rehabilitation). As such, the training given to teachers, doctors and nurses is not producing referrals that are taken seriously by police, and yet the roll-out of Prevent to all social workers continues unabated. It is not considered politically or statistically problematic that the misfire rate of Prevent surveillance in the NHS is 90%.

How can such high numbers of inappropriate referrals be tolerated without necessitating a change in the Prevent Duty? These features can only be treated as unimportant if statistical modelling no longer informs Prevent’s epistemology. The national roll-out of Prevent and the ambivalence towards the 90% misfire rate betrays the presence of a non-statistical epistemology. In big data epistemologies, no data is wasted. Big data analytics privilege large sample sizes because they are understood to reveal patterns of correlation, invisible to the naked eye, from the digital evaluation of unrelated data scraps (Comfort et al. 2010). The processing of both appropriate and inappropriate referrals by local authority panels is, in this paradigm, helpful in the *inductive* generation of terrorism-related patterns and profiles.

It is this epistemological paradigm which informs the merging of the primary (population level) and secondary (high risk groups) categories of pre-crime intervention. The national geographical roll-out of Prevent, which has replaced most implementation in residential communities, involves the radical remodelling of British pre-crime around big data epistemology. A probabilistic model could not recommend the application of secondary measures to the entire population, because probability science is used to isolate particular locations/communities as high risk. The geographical shift in Prevent administration is also epistemological: it has moved away from renderings of “likelihood”, favouring the nationalised roll-out of Prevent training, surveillance and reporting to all corners of the social care system. The imagination of pre-criminal space is now totalising and all encompassing.

## The biopolitics of the prevent duty

In 2008, Michael Dillon and Luis Lobo-Guerrero published an article exploring contemporary biopolitical security, specifically, how security apparatuses have adapted to the molecular age by taking life-itself (rather than species life) as an object to be secured (Dillon and Luis 2008). They introduced modifications to Foucault’s conceptions of biopolitics and security, adapting them to twentieth-century understandings of life and biology. The administration and regulation of life through structures of governance addresses population differently than it once did, moving away from the securitisation of behaviours and economic potentials to imagine insecurity through the contingency of the molecular level (Dillon and Luis 2008, 278). Like much of the literature already explored in this article, Dillon and Lobo-Guerrero show how Foucault’s biopolitics frames the shift from statistical, economic modelling of stable risks to the objectification of contingency as threat (2008, 283–4). Life rendered by medicine and science as pluripotent, they show, provoked a shift in the operations of security such that it now attempts to regulate and bound life’s generative capacity.

This problematisation and securitisation of life-itself is evident in the development of the Prevent Strategy. Counterterrorism has adapted to reconfigured understandings of life and population, moving away from static models of insurgency and rushing to develop to new discourses of radicalisation in the aftermath of the London bombings that could help them to regulate contingency (Kundnani 2015; Sedgwick 2010). That there was felt to be a need for a new counterterrorism discourse to respond to the events is indicative of security’s biopolitical shift towards managing contingency in the twenty-first century. The problematisation of the London bombings, and the 9/11 attacks, as ushering in an era of unpredictability and uncertainty (Rumsfeld 2002) – rather than something that could be understood through studying militant groups and their turn towards attacking the “far enemy” (Gerges 2005) – reflects a concern with the contingency and unpredictability of life.

The reorientation of counterterrorism towards regulating contingency and life (where life is understood as adaptive potential, rather than as species life) is demonstrated most clearly in the re-articulation of counter-radicalisation as safeguarding. Safeguarding procedures are established protocols within social care and health care which make practitioners responsible for noticing, and reporting, physical, sexual and financial abuse of vulnerable people (Home Office 2015b; NHS England 2015). The remaking of Prevent as a safeguarding measure implicitly creates a new type of abuse: ideological abuse (Heath-Kelly 2016). At this point, the structures of care and security become blurred beyond distinction – revealing their common biopolitical heritage as structures productive and governing of population (Howell 2014).

Under the Conservative governments, pre-criminal space has been reimagined as a totalising geography. The radical contingency of each life is interpreted as uniquely dangerous, requiring of a population-wide system of monitoring for unauthorised adaptations. I have referred to this elsewhere (Heath-Kelly 2016) as the autoimmune moment in British security policy where the distinction between suspicious and non-suspicious bodies has collapsed; in the absence of traditional immunological security politics, the surveillance of all life, in totality, is now understood as biopolitically necessary and advantageous.

It would befit the future of the Critical Terrorism Studies project to study the intertwining of social care structures with counterterrorism, as well as the blurring of digital and non-digital epistemologies of surveillance and calculation.

## Conclusion

This article has explored the shifting geography and epistemology of the Prevent strategy from 2007 until 2017. It has highlighted the de-emphasising of residential communities as sites of intervention during the Coalition and Conservative periods, arguing that this reveals the shift from calculative rationalities of probability towards big data logics of inductive profiling.

In Labour’s Prevent Strategy, the tripartite typology of primary, secondary and tertiary crime prevention measures informed the delivery of counter-radicalisation. Local authorities became important players in the deployment of Prevent’s secondary-level measures intended to build “resilience” to extremism in “vulnerable” communities. But rather than statistically modelling those communities “most probable” to produce terrorists from available data (a task for which probabilistic science is currently incapable (Sageman 2016)), Labour governments operationalised race as the foundation for their imagination of pre-criminal space. Discourses of radicalisation re-signified British Asian communities as areas of higher extremism risk, and those communities were then targeted with crime prevention measures.

After the change in government, the Prevent review severed the DCLG from Prevent strategy operations and criticised Labour’s blurring of integration and counterterrorism. This criticism of Prevent’s secondary level of intervention did not end community profiling entirely, however. Some targeted local authorities still deliver some Central Government produced workshops and events, even though the deployment of Prevent through communities is reduced. Instead, the Prevent Duty has now radically altered the geography of counter-radicalisation. Prevent is now nationally deployed across the educational and healthcare sectors. This geographical shift embodies the merging of the primary and secondary levels of prevention: whole-of-population measures have been merged with those identifying more vulnerable groups. The two levels of intervention have merged.

This epidemiological revolution betrays a shift in the epistemology of Prevent. The massive roll-out of Prevent training to all NHS and educational staff does not respond to a population-wide insurgency, nor the likelihood that schools and hospitals are being used as bases for conspiracy. Rather, the educational and health-care sectors have been incorporated into counterterrorism because they have significant access to the public. Home Office documentation lauds the prolific patient contacts experienced by the NHS, arguing that this makes it a valid partner in counterterrorism.

The value accorded to scale betrays a move away from probabilistic science. Algorithmic and big data logics have become prominent within digital surveillance and employ huge data sets in their modalities. The calculative discourse associated with the big data paradigm (more data leads to the identification of previously hidden patterns and detections) appears to have influenced the geographic shift in Prevent’s deployment (in addition to the economic austerity drive of the Conservative and Coalition governments). Otherwise, the geographical shift to nationalised Prevent surveillance in all social care facilities would make little sense. Furthermore, the huge rejection rates of Channel referrals by the police would not be tolerated under a probabilistic paradigm.

To conclude, the British government seems to be arriving at the conclusion (qua Marc Sageman) that terrorism cannot be statistically predicted in the style of other crimes. Instead, the fluctuations of the Prevent strategy under varying British governments provide a window onto the biopolitical constitution of pre-criminal geographies, where the contingency of life itself demands regulation, management and intervention. The imagination of pre-criminal space has extended outwards, in a dramatic colonisation of social care by counterterrorism.

## Notes

1. Safeguarding is the practice of protecting children and adults (with care needs) from financial, physical and sexual abuse. The duty to note concerns about abuse and escalate them is incumbent upon school and health-care staff. The Prevent Duty has inserted a new category of abuse, “radicalisation”, into safeguarding protocol (Home Office 2015b; NHS England 2015).

2. In 2015, it became a legal duty for health-care providers, schools and universities to take part in the suppression of “radicalisation” and report subjects of concern to the police. In schools and health care, this is framed as a safeguarding duty performed for the benefit of the subject concerned.

3. In the decades prior to 9/11, much terrorism research was concerned with the root structural causes of terrorism, or locating the connections between protest cycles and violent offshoot groups, or mapping the pathological mindset which enables terrorist crimes.

4. The Terrorism Prevention and Investigatory Measures Act replaced Control Orders in 2011. They enforce a curfew upon a suspect and limit their use of phones and internet.

5. However, tertiary-level prevention continues through police-led Channel panels in each local authority.

## Appendix

**Table:** Priority local authorities which received funding from the Prevent Pathfinder Fund.

Region	Priority local authorities
South West	Bristol City Council
South East	Wycombe District Council; Oxford City Council; Reading Borough Council; Royal Borough of Windsor and Maidenhead; Slough Borough Council; Crawley Borough Council; Woking Borough Council
London	Barking and Dagenham Council; London Borough of Barnet; Brent Council; Camden Council; London Borough of Croydon; Ealing Council; Enfield Council; Greenwich Council; London Borough of Hackney; Hammersmith and Fulham Council; Haringey Council; Harrow Council; London Borough of Hillingdon; Hounslow Council; Islington Council; Royal Borough of Kensington and Chelsea; London Borough of Lambeth; Lewisham Council; London Borough of Merton; London Borough of Newham; London Borough of Redbridge; Southwark Council; Tower Hamlets Council; London Borough of Waltham Forest; Wandsworth Borough Council; City of Westminster City Council
East of England	Bedford Borough Council; Luton Borough Council; Peterborough City Council; Watford Borough Council
East Midlands	Derby City Council; Leicester City Council; Nottingham City Council
West Midlands	Birmingham City Council; Dudley; Metropolitan Borough Council; Sandwell Metropolitan Borough Council; Stoke-on-Trent City Council; Walsall Council
Yorkshire and the Humber	Bradford Metropolitan District Council; Calderdale Council; Kirklees Council; Leeds City Council; Wakefield City Council
North West	Bolton Council; Bury Metropolitan Borough Council; Manchester City Council; Oldham Metropolitan Borough Council; Rochdale Metropolitan Borough Council; Salford City Council; Stockport Metropolitan Borough Council; Tameside Metropolitan Borough Council; Trafford Council; Wigan Council; Blackburn with Darwen Borough Council; Burnley Borough Council; Hyndburn Borough Council; Pendle Borough Council; Preston City Council; Ribble Valley Borough Council; Rossendale Borough Council
North East	Middlesbrough Borough Council; Newcastle City Council

Source: Department of Communities and Local Government 2007 (14–5).
